# Development of machine learning models for the detection of surgical site infections following total hip and knee arthroplasty: a multicenter cohort study

**DOI:** 10.1186/s13756-023-01294-0

**Published:** 2023-09-02

**Authors:** Guosong Wu, Cheligeer Cheligeer, Danielle A. Southern, Elliot A. Martin, Yuan Xu, Jenine Leal, Jennifer Ellison, Kathryn Bush, Tyler Williamson, Hude Quan, Cathy A. Eastwood

**Affiliations:** 1 Department of Community Health Sciences, Cumming School of Medicine, University of Calgary, Calgary, AB Canada; 2 The Centre for Health Informatics, Cumming School of Medicine, University of Calgary, Calgary, AB Canada; 3 Alberta Health Services, Calgary, AB Canada; 4 Departments of Oncology, Community Health Sciences, University of Calgary, Calgary, AB Canada; 5 Departments of Surgery, Cumming School of Medicine, University of Calgary, Calgary, AB Canada; 6 Infection Prevention and Control Surveillance and Standards, Alberta Health Services, Calgary, AB Canada; 7 Department of Microbiology, Immunology, and Infectious Diseases, Cumming School of Medicine, University of Calgary, Calgary, AB Canada; 8 O’Brien Institute for Public Health, University of Calgary, Calgary, AB Canada; 9 AMR-One Health Consortium, University of Calgary, Calgary, AB Canada

**Keywords:** Surgical site infections, Total hip arthroplasty, Total knee arthroplasty, Machine learning

## Abstract

**Background:**

Population based surveillance of surgical site infections (SSIs) requires precise case-finding strategies. We sought to develop and validate machine learning models to automate the process of complex (deep incisional/organ space) SSIs case detection.

**Methods:**

This retrospective cohort study included adult patients (age ≥ 18 years) admitted to Calgary, Canada acute care hospitals who underwent primary total elective hip (THA) or knee (TKA) arthroplasty between Jan 1st, 2013 and Aug 31st, 2020. True SSI conditions were judged by the Alberta Health Services Infection Prevention and Control (IPC) program staff. Using the IPC cases as labels, we developed and validated nine XGBoost models to identify deep incisional SSIs, organ space SSIs and complex SSIs using administrative data, electronic medical records (EMR) free text data, and both. The performance of machine learning models was assessed by sensitivity, specificity, positive predictive value, negative predictive value, F1 score, the area under the receiver operating characteristic curve (ROC AUC) and the area under the precision–recall curve (PR AUC). In addition, a bootstrap 95% confidence interval (95% CI) was calculated.

**Results:**

There were 22,059 unique patients with 27,360 hospital admissions resulting in 88,351 days of hospital stay. This included 16,561 (60.5%) TKA and 10,799 (39.5%) THA procedures. There were 235 ascertained SSIs. Of them, 77 (32.8%) were superficial incisional SSIs, 57 (24.3%) were deep incisional SSIs, and 101 (42.9%) were organ space SSIs. The incidence rates were 0.37 for superficial incisional SSIs, 0.21 for deep incisional SSIs, 0.37 for organ space and 0.58 for complex SSIs per 100 surgical procedures, respectively. The optimal XGBoost models using administrative data and text data combined achieved a ROC AUC of 0.906 (95% CI 0.835–0.978), PR AUC of 0.637 (95% CI 0.528–0.746), and F1 score of 0.79 (0.67–0.90).

**Conclusions:**

Our findings suggest machine learning models derived from administrative data and EMR text data achieved high performance and can be used to automate the detection of complex SSIs.

**Supplementary Information:**

The online version contains supplementary material available at 10.1186/s13756-023-01294-0.

## Highlights

- The incidence rates of surgical site infections following total hip and knee arthroplasty were 0.5 and 0.52 per 100 surgical procedures.
- The incidence of SSIs varied significantly between care facilities (ranging from 0.53 to 1.71 per 100 procedures).
- The optimal machine learning model achieved a ROC AUC of 0.906 (95% CI 0.835–0.978), PR AUC of 0.637 (95% CI 0.528–0.746), and F1 score of 0.79 (0.67–0.90).

**Supplementary Information:**

The online version contains supplementary material available at 10.1186/s13756-023-01294-0.

## Background

Surgical site infections (SSIs) are one of the most common healthcare-associated infections (HAIs) in post-operative procedures [1]. In North America, SSIs occur in 2–5% of all surgeries and are associated with extended hospital stays of 11 days, resulting in an increased care cost of 13,000 USD per patient admission [2, 3]. The SSI rate varies significantly, ranging from 0.6 to 9.5%, depending on the type of surgical procedure, as reported in the European Centre for Disease Prevention and Control’s (ECDC) 2023 surveillance report [4]. Patients with SSIs are more likely to be admitted to critical care units and have a five-fold increase in hospital readmissions [2]. About 77% of surgical patient deaths are associated with SSIs [2].

By the next decade, the demand for total hip (THA) and knee (TKA) arthroplasty procedures in the US is projected to grow by 174% and 673%, respectively [5]. While many infection prevention and control (IPC) strategies are implemented in clinical practice (e.g., improved ventilation in operating rooms, sterilization methods, surgical techniques, antibiotic prophylaxis), SSIs remain a substantial cause of adverse patient outcomes [6]. Surveillance programs audit the occurrence of SSIs. Identifying SSIs from large population-based databases can improve the completeness, accuracy, and efficiency of SSI surveillance programs [7]. In Canada, SSI case identification relies on International Classification of Diseases (ICD) codes [8], sometimes followed by a comprehensive chart review to confirm the presence of SSIs [7, 9]. As such, traditional surveillance methods rely on manual chart review by trained reviewers. This process is time-consuming, labour-intensive, and expensive. Additionally, it is well-studied that administrative data-based adverse event detection methods are suboptimal due to under-coding or miss-coding [10].

Electronic medical records (EMR) have been widely implemented and contain detailed and comprehensive information regarding all aspects of patient care, offering a valuable complement to coded data. The advance of artificial intelligence technologies promoted research on free text data, which enabled analysis of large, complex EMR text data sets. Machine learning models employed on EMR free-text data can significantly improve the detection of SSIs [11]. The purpose of this study was to determine the incidence of SSI and to develop machine learning models to automate the process of detecting complex (deep incisional/ organ space) SSI following THA/TKA.

## Methods

### Patient cohort

We included adult patients (age ≥ 18 years) who were admitted to any tertiary acute care hospitals in Calgary, Canada, and underwent primary total elective hip or knee arthroplasty between January 1st, 2013, and August 31st, 2020. Patients who underwent hemiarthroplasty, cement spacers, revisions, or abandoned procedures were excluded.

### Data sources

The study cohort was defined using the Canadian Classification of Health Interventions (CCI) administrative codes documented in the Alberta Discharge Abstract Database (DAD), with up to 20 procedure codes per record [12]. Patient information was pulled if any of the following CCI codes were documented in their records: 1.VA.53 (Implantation of internal device, hip joint), 1.SQ.53 (Implantation of internal device, pelvis), 1.VG.53 (Implantation of internal device, knee joint), 1.VP.53 (Implantation of internal device, patella) [13]. Structured data such as patient demographic information, diagnosis codes (up to 25 ICD 10th revision in Canada [ICD-10-CA] codes), procedure details and patient outcomes were extracted.

Sunrise Clinical Manager (SCM) is an inpatient electronic medical record system being used at the time of this study in all Calgary hospitals. SCM EMR captures demographic, clinical, and outcome data for all patients admitted to the study hospitals. To develop machine learning models, we extracted the free text data of nursing notes for patients who were readmitted to the Calgary hospitals within 90 days following a THA or TKA procedure. The patient's personal healthcare number (PHN) and unique lifetime identifiers (ULI) were used to link data sets. Patient records without valid PHNs or ULIs were excluded.

## Reference standard

Surgical site infection (SSI) is defined by the Centers for Disease Control and Prevention (CDC) as an infection that occurs after surgery in the part of the body where the surgery took place [14]. True SSI conditions were identified by trained Alberta Health Services’ IPC program staff through a provincial SSIs surveillance program using a priorly established methodology [7]. This comprehensive process includes reviewing patient microbiology laboratory results, patient charts, re-operation records, readmissions, emergency visit records, and clinic visit records. The National Healthcare Safety Network definitions are used to classify superficial incisional SSI, deep incisional SSI or organ/space SSI [15]. Complex SSIs are those classified as deep incisional or organ/space SSI. Manual case detection is supplemented with an administrative linkage using ICD-10 codes to increase case detection [7]. Since mandatory reporting of superficial SSIs was terminated in April 2018 in Alberta, the incidence rate of superficial SSIs was calculated using data collected before April 2018. In our reference data set, all patients were followed for 90 days after the surgical procedure date to observe if they developed infections. The results from this review served as the reference standard for developing and validating the machine learning models.

### Data preprocessing and feature extraction

The proposed method composed of both structured and unstructured datasets. Please refer to the Additional file 1 for information concerning data properties and the specifics of model development. After linking all datasets, using the reference standard data we created a variable for ‘Not infected,’ ‘Organ-space infection,’ or ‘Deep incisional infection’. To build a structured dataset, we extracted all unique ICD-10 codes from DAD for the patient cohort to serve as main features and used one-hot encoding to represent each patient [16]. The application of this technique yielded a feature matrix that is sparse in nature. In this matrix, each row corresponds to a patient’s hospital stay, while each column represents a unique ICD code. By leveraging this approach, we were able to efficiently represent patient data in a concise format, which will be passed to the downstream machine learning model together with text dataset.

For the text dataset construction, we choose Multidisciplinary Progress Report (MPR) from each patient’s EHR from the database of SCM EMR. An MPR is a nursing note that summarizes the nursing care plan and the patient’s treatment response over a period of time. It also containing patient’s vital sign, medication administration, nursing intervention, and any changes to patients condition. The MPRs for our cohort were pre-processed with the following techniques in sequence: case folding, lemmatization, stopwords removal, special character handling, medical concept extraction, and negation detection [16, 17]. To analyze text, we use a method called Bag-of-Words (BOW) that converts text into feature vectors, where each position in the vector represents the occurrence of the frequency of unique word or phrase from the text [16]. Then, we employed the term frequency–inverse document frequency (TF–IDF) weighting models to enhance the characterization of significant words in BOW representation. The resulting TF–IDF scores provided a more robust measure of word importance in the analyzed health informatics documents [16]. Once we have the TF–IDF feature matrix, we concatenate it with the ICD-10 feature matrix to get a merged representation for patient cohort. After the feature extraction, the dataset was split into training and testing sets by an 80:20 ratio.

### Model development

**Fig. 1:**
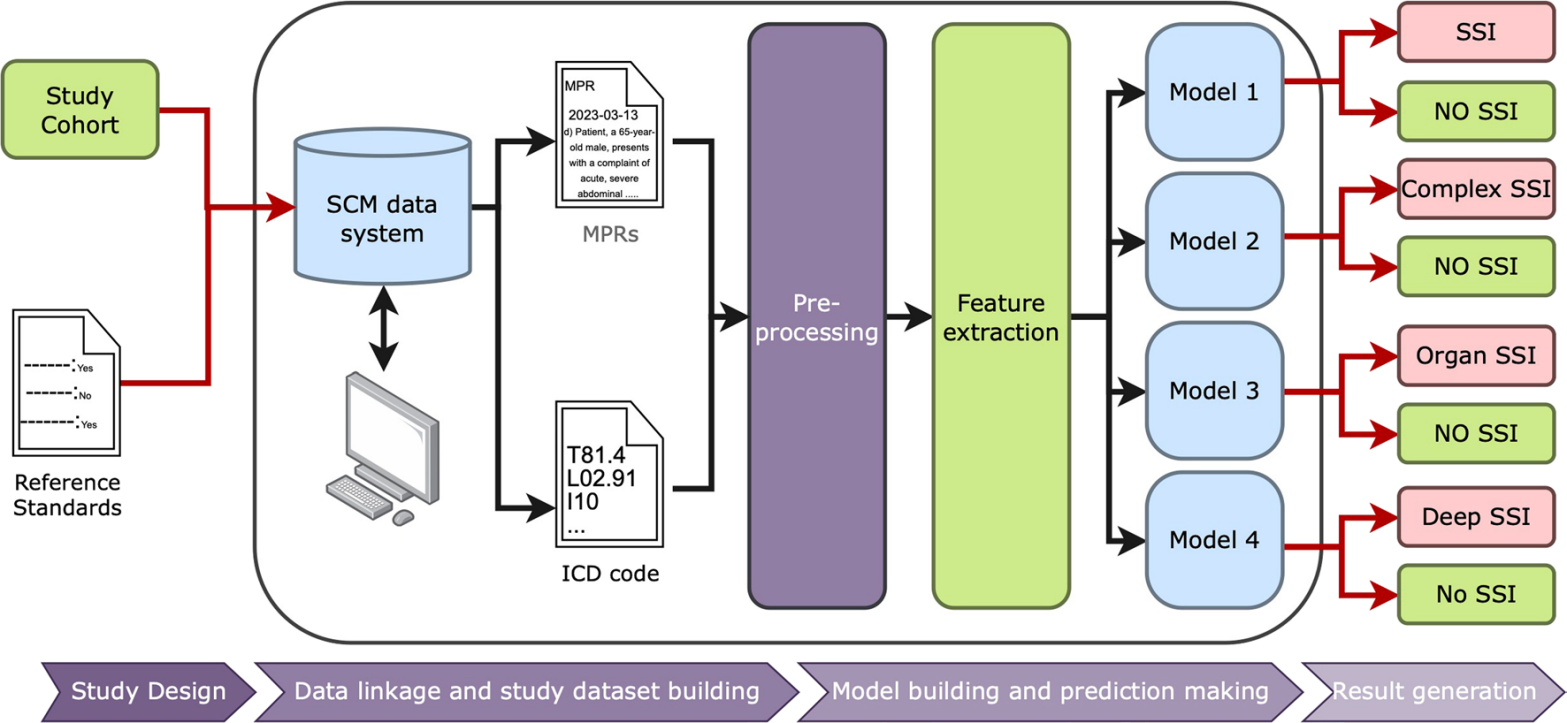
Schematic Representation of Data Linkage and ML Model for SSI Detection. *MPRs* multiplanary progress report, *SCM* sunrise clinical manager, *SSI* surgical site infections

We developed nine XGBoost models to identify deep incisional SSI, organ/space SSI and complex SSI using administrative data, EMR free text data and both types of data. XGBoost is a machine learning model that combines weak decision trees to perform regression and classification. To optimize the performance of the XGBoost model, we performed a grid search using the GridSearchCV function from the Scikit-learn library [18]. The grid search involved creating a range of hyperparameter values, training the model for each combination of hyperparameters, and evaluating its performance using cross-validation and a specified scoring metric. Hyperparameters (e.g., learning_rate, max_depth, gamma, reg_lambda, etc.) were tuned to maximize models’ sensitivity. Optimal hyperparameters were utilized for training our final XGBoost models. Fine-tuned XGBoost models were evaluated using the preserved testing sets. For a detailed illustration of our methodology, please refer to Fig. 1.

### Statistical analysis

Patient demographic and clinical characteristics were summarised using frequencies and percentages or medians and interquartile ranges (IQRs) as appropriate. The Charlson Comorbidity Index (CCI) was calculated for each patient based on their 25 diagnosis codes documented in the DAD using the weighted score approach [19]. Chi-square tests and Wilcoxon rank-sum tests analyzed the comparison of categorical variables. Performance of SSI machine learning models was assessed by sensitivity, specificity, positive predictive value (PPV), negative predictive value (NPV), and F1 score. We computed the area under the receiver operating characteristic curve (ROC AUC) to evaluate the trade-off between the sensitivity and specificity of the XGBoost models on various thresholds. In this study, the occurrence of SSIs is significantly lower in comparison to those without infections, resulting in imbalanced data, which can present challenges during the evaluation of machine learning algorithms. The area under the precision–recall curve (PR AUC) was computed to present an average precision that combines PPV and sensitivity in a single visualization. Unlike the ROC AUC baseline of 0.5 (random classifier), the PR AUC baseline is the fraction of positives among the total sample. Different classes have different baselines. The PR AUC is a powerful performance measure for imbalanced data when the incidence of SSI is low and to identify positive SSI cases with minimal false positives [20]. The Scikit-learn Python library was used for AUC statistics, and a bootstrap 95% confidence interval (95% CI) was calculated. XGBoost Python library was used for model development, and the Imbalanced-learn library was applied for resampling training data. All statistical analyses were performed using Stata 16 software (StataCorp. 2019. *Stata Statistical Software: Release 16*. College Station, TX: StataCorp LLC.) and Python 3.10 [21].

## Results

### Study population

The study cohort consisted of 22,059 unique patients with 27,360 hospital admissions resulting in 88,351 days of hospital stay. This included 16,561 (60.5%) TKA and 10,799 (39.5%) THA procedures (Table 1). The median age was 66 years (IQR 59–73), 43.26% were male, and 96.6% were comorbidity-free. The patients spent a median of three days (IQR 2–4) in hospital at the time of the TKA and THA procedure, most of whom were discharged home.

**Table 1:** Characteristics of patients who underwent primary total elective hip or knee arthroplasty, 2013–2020

Characteristic	Overall cohort (n = 27,360)	SSIs (n = 235)	No SSIs (n = 27,125)	*p*-value
Age (years), median (IQR)	66 (59–73)	66 (59–72)	66 (59–73)	0.686†
Male	11,836 (43.26)	129 (54.89)	11,707 (43.16)	< 0.001‡
Charlson comorbidity index				0.258‡
0	23,616 (86.32)	195 (82.98)	23,421 (86.34)	
1	2691 (9.84)	27 (11.49)	2664 (9.82)	
> 2	1053 (3.85)	13 (5.53)	1040 (3.83)	
Procedure type				0.001‡
Knee implantation	16,561 (60.53)	16,419 (60.53)	142 (60.43)	
Hip implantation	10,799 (39.47)	10,706 (39.47)	93 (39.57)	
ICU admission	33 (0.12)	1 (0.43)	32 (0.12)	0.176‡
Length of hospital stay, median (IQR)	3 (2,4)	3 (2,5)	3 (2,4)	0.001†
Discharge disposition				0.012‡
Home	26,109 (95.43)	221 (94.04)	25,888 (95.44)	
Transfer	882 (3.22)	10 (4.26)	872 (3.21)	
Long term care	11 (0.04)	0	11 (0.04)	
Death	13 (0.05)	0	13 (0.05)	

*IQR* interquartile range, *SSIs* surgical site infections

^†^Wilcoxon rank-sum test;

^‡^Chi-square test

### SSIs description

Among all observed procedures, 17,991 were performed before April 2018, and 9,369 were performed after. The chart review ascertained 235 SSIs, resulting in an overall incidence rate of 0.86 per 100 surgical procedures. Of them, 77 (32.8%) were superficial incisional SSIs (66 of which occurred before 2018), 57 (24.3%) were deep incisional SSIs, 101 (42.9%) were organ space SSIs, and 158 (67.2%) were complex SSIs. The incidence rates were 0.37 for superficial incisional SSIs, 0.21 for deep incisional SSIs, 0.37 for organ space SSIs and 0.58 for complex SSIs per 100 surgical procedures.

Specifically, a total of 16,370 TKA were observed, with 10,914 performed before April 2018. Chart review confirmed a total of 138 SSIs, comprising 54 (39.1%) superficial incisional SSIs (45 of which occurred before 2018), 34 (24.6%) deep incisional SSIs, 50 (36.3%) organ space SSIs, and 84 (60.9%) complex SSIs. The corresponding incidence rates were 0.41 for superficial incisional SSIs, 0.21 for deep incisional SSIs, 0.31 for organ space SSIs, and 0.52 for complex SSIs per 100 surgical procedures. Among the total 10,990 THA procedures observed, 7,077 were performed before April 2018. Through chart review, we identified 23 (23.7%) superficial incisional SSIs (21 occurring before 2018), 23 (23.7%) deep incisional SSIs, 51 (52.6%) organ space SSIs, and 74 (76.3%) complex SSIs. The respective incidence rates were 0.3 for superficial incisional SSIs, 0.21 for deep incisional SSIs, 0.29 for organ space SSIs, and 0.5 for complex SSIs per 100 surgical procedures.

SSIs incidence varied significantly between hospitals (ranging from 0.53 to 1.71 per 100 procedures). A significant decrease was observed in the incidence of SSIs over the study period (incidence rate ratios [IRR] per year 0.93; 95% CI 0.87–0.98).

Table 2 describes the nature of SSIs in this study cohort. The median age of patients with an SSI was 66 (IQR 59–72), and 54.9% were male. Blood culture tests were positive for only 29.9% of superficial incisional SSIs but increased to 87.7% of deep incisional SSIs and 98.0% of organ space SSIs.

**Table 2:** Characteristics of patients with surgical site infections following total hip or knee arthroplasty, 2013–2020

Characteristic	Overall Cohort (n = 235)	Superficial SSIs (n = 77)	Deep SSIs (n = 57)	Organ space SSIs (n = 101)	*p*-value
Age (years), median (IQR)	66 (59–72)	64 (58–75)	67 (60–71)	65 (59–72)	0.879†
Male	129 (54.89)	34 (44.16)	37 (64.91)	58 (57.43)	0.046‡
Charlson comorbidity index					0.244‡
0	195 (82.98)	65 (84.42)	48 (84.21)	82 (81.19)	
1	27 (11.49)	11 (14.29)	6 (10.53)	10 (9.90)	
> 2	13 (5.53)	1 (1.30)	3 (5.26)	9 (8.91)	
Procedure type					0.021‡
Knee implantation	138 (58.72)	54 (70.13)	34 (59.65)	50 (49.50)	
Hip implantation	97 (41.28)	23 (29.87)	23 (40.35)	51 (50.50)	
Procedure lateral					0.595‡
Left	118 (50.21)	37 (48.05)	32 (56.14)	49 (48.51)	
Right	111 (47.23)	37 (48.05)	25 (43.86)	49 (48.51)	
Bilateral	6 (2.55)	3 (3.90)	0	3 (2.97)	
Blood culture type					< .0001‡
Superficial wound swab	22 (11.58)	19 (50.00)	2 (3.92)	1 (0.99)	
Deep wound	39 (20.53)	2 (5.26)	22 (43.14)	15 (14.85)	
Tissue	82 (43.16)	6 (15.79)	17 (33.33)	59 (58.42)	
Fluid aspirate	35 (18.42)	10 (26.32)	8 (15.69)	17 (16.83)	
Other	12 (6.32)	1 (2.63)	2 (3.92)	9 (8.91)	
Blood culture result					< .0001‡
Positive	172 (73.19)	23 (29.87)	50 (87.72)	99 (98.02)	
Negative	18 (7.66)	15 (19.48)	1 (1.75)	2 (1.98)	
NA	45 (19.15)	39 (50.65)	6 (10.53)	0	
Days from admission to procedure	0	0	0	0	–
Days from procedure to discharge	3 (2,5)	3 (3,4)	3 (2,5)	3 (2,5)	0.928†
Length of hospital stay, median, days	3 (2,5)	3 (3,4)	3 (2,5)	3 (2,5)	0.940†
Discharge disposition					0.138‡
Home	221 (94.04)	72 (93.51)	51 (89.47)	98 (97.03)	
Transfer	10 (4.26)	4 (5.19)	3 (5.26)	3 (2.97)	
Death/long term care	0	0	0	0	
Re-admission	201 (85.53)	45 (58.44)	57 (100.00)	99 (98.02)	< .0001‡

*IQR* interquartile range, *SSIs* surgical site infections

^†^Wilcoxon rank-sum test;

^‡^Chi-square test

### Performance of machine learning models

**Fig. 2:**
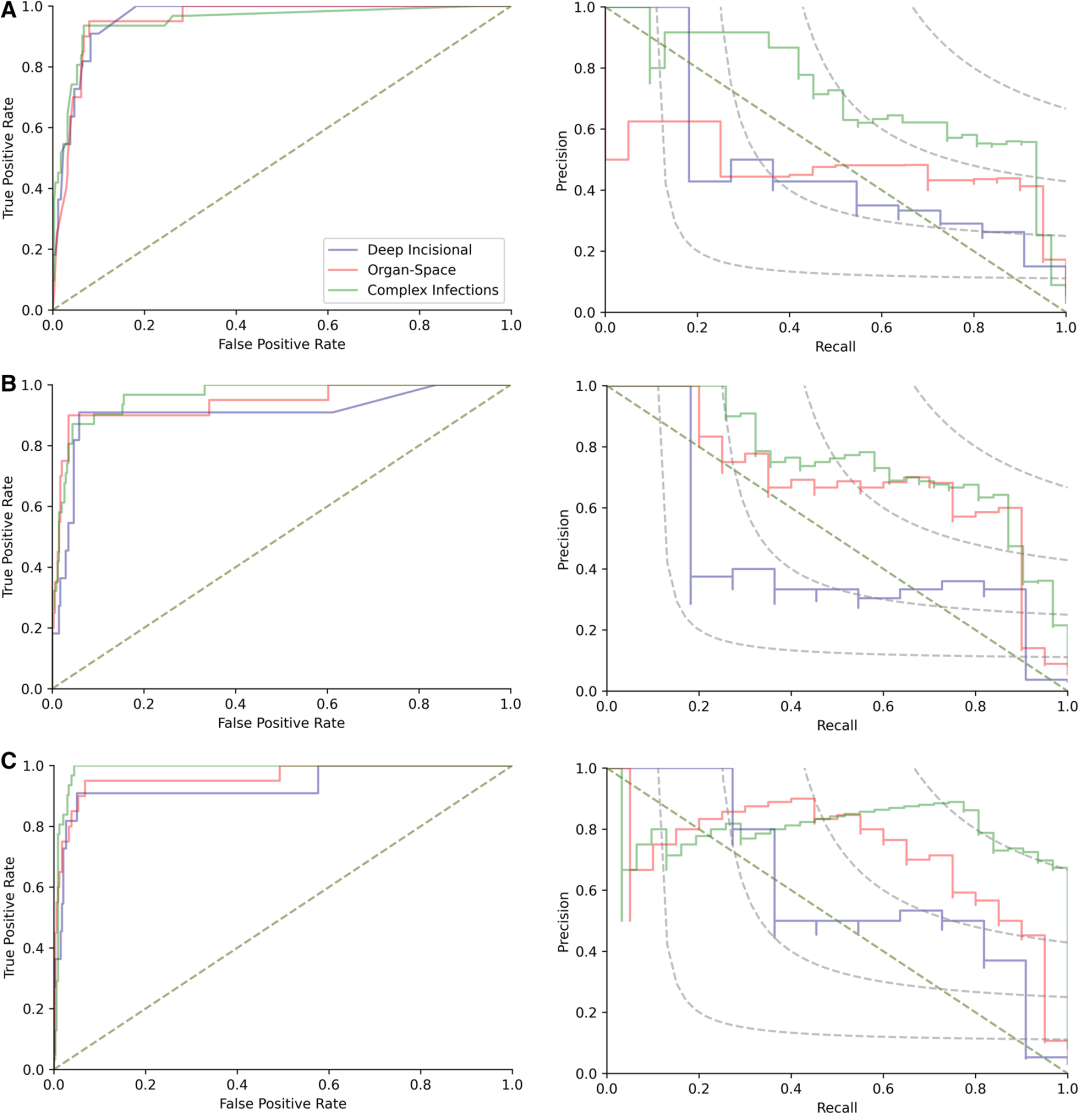
Performance of XGBoost models for the detection of surgical site infections. **A** The area under the receiver operating characteristic curves (ROC AUC, left) and the area under precision–recall curves (PR AUC, right) for the administrative data based XGBoost models. **B** The area under the receiver operating characteristic curves (ROC AUC, left) and the area under precision–recall curves (PR AUC, right) for the EMR text data based XGBoost models. **C** The area under the receiver operating characteristic curves (ROC AUC, left) and the area under precision–recall curves (PR AUC, right) for the mix using of administrative and text data based XGBoost models

Overall, the XGBoost models using a combination of administrative data and text data to identify complex SSIs achieved the best performance, with an F1 score of 0.788, ROC AUC of 0.906 (95% CI 0.835–0.978), and PR AUC of 0.637 (95% CI 0.528–0.746) (Table 3). Compared with models derived from administrative data, the models derived from text data had a higher F1 score (0.735 vs. 0.699), PR AUC (0.561 [95% CI 0.452–0.67] vs. 0.527 [95% CI 0.419–0.635]) and PPV (67.5% [95% CI 53.8–78.9%] vs. 55.8% [95% CI 45.7–65.4]) in identifying complex SSIs (Fig. 2), but a lower ROC AUC (0.886 [0.808–0.963] vs. 0.934 [0.873–0.995]) and sensitivity (80.7 [95% CI 62.5–92.6%] vs. 93.6 [95% CI 78.6–99.2%]).

**Table 3:** Performance measures for developed machine learning algorithms for the detection of SSIs

Algorithms	F1 score	ROC AUC (95% CI)	PR AUC (95% CI)	Sensitivity% (95% CI)	Specificity% (95% CI)	PPV % (95% CI)	NPV% (95% CI)
Admin data algorithms							
Deep SSIs	0.421 (0.214–0.605)	0.836 (0.687–0.984)	0.224 (0.114–0.334)	72.73 (39.03–93.98)	94.41 (91.41–96.60)	29.63 (19.27–42.61)	99.07 (97.60–99.65)
Organ SSIs	0.552 (0.385–0.693)	0.868 (0.765–0.97)	0.348 (0.237–0.459)	80.00 (56.34–94.27)	93.51 (90.34–95.89)	42.11 (31.47–53.52)	98.75 (97.06–99.48)
Complex SSIs	0.699 (0.575–0.805)	0.934 (0.873–0.995)	0.527 (0.419–0.635)	93.55 (78.58–99.21)	93.24 (90.02–95.66)	55.77(45.67–65.41)	99.37 (97.65–99.84)
Text data algorithms							
Deep SSIs	0.414 (0.174–0.640)	0.755 (0.587–0.923)	0.196 (0.967–0.296)	54.55 (23.38–83.25)	96.47 (93.92–98.16)	33.33 (18.73–52.03)	98.50 (97.17–99.21)
Organ SSIs	0.638 (0.439–0.794)	0.857 (0.752–0.962)	0.431 (0.307–0.554)	75.00 (50.90–91.34)	96.46 (93.90–98.16)	55.56 (40.43–69.71)	98.49 (96.84–99.29)
Complex SSIs	0.735 (0.607–0.848)	0.886 (0.808–0.963)	0.561 (0.452–0.67)	80.65 (62.53–92.55)	96.47 (93.92–98.16)	67.57 (53.80–78.85)	98.20 (96.38–99.12)
Admin& Text algorithms							
Deep SSIs	0.500 (0.222–0.714)	0.762 (0.596–0.929)	0.266 (0.142–0.39)	54.55 (23.38–83.25)	97.94 (95.80–99.17)	46.15 (25.65–68.05)	98.52 (97.21–99.22)
Organ SSIs	0.714 (0.522–0.857)	0.865 (0.762–0.968)	0.525 (0.393–0.657)	75.00 (50.90–91.34)	97.94 (95.79–99.17)	68.18 (49.66–82.31)	98.52 (96.88–99.30)
Complex SSIs	0.788 (0.667–0.896)	0.906 (0.835–0.978)	0.637 (0.528–0.746)	83.87 (66.27–94.55)	97.35 (95.03–98.78)	74.29 (59.82–84.86)	98.51 (96.74–99.33)

*95% CI* 95% confidence interval, *NPV* negative predictive value, *PPV* positive predictive value, *PR AUC* the area under the precision–recall curve, *ROC AUC* the area under the receiver operating characteristic curve, *SSIs* surgical site infections

## Discussion

In this population-based multicenter cohort study, we observed a modestly reduced incidence of SSIs following total hip and knee arthroplasty over the study period, in contrast to the findings reported in existing literature. The incidence of SSIs varied substantially across hospitals. We developed and evaluated nine machine learning models to identify SSIs from patient charts. The model that was developed using both structured and unstructured (nursing notes) data achieved the best performance. Applying these models has the potential to reduce the workload for chart reviews of traditional IPC surveillance programs.

Surveillance and reporting of SSIs are critically important to prevent and control healthcare-associated infections. Parameters such as data quality of different surveillance programs, postsurgical follow-up process and imperfect criteria potentially contribute to the discordance of reported incidence of SSIs in literature [22]. In our study, the SSI rates for TKA and THA were 0.52% and 0.5%, respectively. Comparatively, the CDC reported rates for TKA and THA were 0.65% and 0.4%, and the ECDC rates were 0.6% and 1.2%, respectively [4, 23]. While our study’s TKA and THA rates were slightly lower than the CDC and ECDC reported rates [2, 24, 25]. This finding is consistent with previously published studies [26]. The observed decrease in the incidence of SSIs throughout the study period might have resulted from uniform provincial surveillance initiated by the Alberta Health Services IPC program starting in March 2012 [27].

The detection of SSIs from large population-based cohorts is shifting from solely relying on the composition of ICD codes to a mixed-use of patient structured and unstructured data leveraging the advantages of machine learning techniques [11]. Clinical notes often contain valuable unstructured textual diagnoses and important clinical events, and have demonstrated enormous benefits for enhancing machine learning models` performance. For example, Bucher et al. developed a natural language processing approach using clinical notes to automate SSI surveillance [28]. As a result, they reached a sensitivity of 0.79 and ROC AUC of 0.852 in their external validation model. In our study, the optimal model achieved a sensitivity of 83.9% (95% CI 66.3–94.6%), ROC AUC of 0.906 (95% CI 0.835–0.978), PR AUC of 0.637 (95% CI 0.528–0.746) and F1 score of 0.79. Adding nursing notes in model development improved our model's general performance, with an increase in the F1 score from 0.699 to 0.788 and an increase in PR AUC from 0.52 to 0.64. Considering the comparison baseline of PR AUC is the incidence of SSI, the magnitude of improvement is substantial.

Our study highlighted that a standard text description structure of nursing notes in EMR could potentially improve the accuracy of SSI detection models. For example, describe the observed evidence of SSIs (e.g., intraoperative cultures, purulent drainage, blood culture test positive, etc.) and conclude that its presence in notes would dramatically improve the possibility of machines in identifying SSIs from the text patterns.

Our findings demonstrate that accurate machine learning models can be developed using administrative and EMR text data. Three sets of models developed from this study can be easily translated into surveillance programs. For example, the set of models could be a tool for an initial screening patient charts to locate the most likely SSIs or exclude the negative cases, saving time and cost to enable large population-based surveillance. The developed models could also be applied to clinical practice to support quality improvement initiatives locally, nationally, or internationally. We believe that the developed models hold the potential to effectively decrease the workload of SSI surveillance, and determining the extent of this reduction represents a valuable direction for future research.

The generalizability of our models to other hospitals is a critical consideration. While the models demonstrated promising results in our specific setting, their applicability to other healthcare facilities may vary. The success of the models largely depends on the availability and quality of data in each hospital's EMR system. Therefore, rigorous validation and customization are strongly recommended before deploying our models in other settings to ensure their accuracy and effectiveness within the unique context of each hospital's healthcare environment.

Finally, while our model has shown promise, there is room for improvement, particularly in terms of precision and reliability. For instance, employing more advanced representations of data, such as language models and embeddings for text data, could be particularly beneficial. Techniques such as transformer-based models like BERT or GPT have shown a remarkable ability to understand the nuanced context within the text and can convert text into high-dimensional vectors, or embeddings, that encapsulate semantic meaning. Utilizing these advanced techniques in our models represents a significant area for future improve our ability to detect SSIs.

### Limitations

Our study had several limitations. First, the reported incidence rates of SSIs were calculated using 90 days of follow-up as literature suggests most SSIs tend to occur within the first 3 months following surgery [7, 14, 29]. Different follow-up days may generate discordance in SSI incidence rates. While using restricted follow-up days (e.g., 30 or 60 days) may improve the precision of models, the sensitivity will be compromised. Researchers need to choose the cut-offs according to their research objectives. Second, the imbalanced data may create challenges for machines to capture the text patterns of SSI cases. We employed random over sampling strategies during the model training phase to improve the performance of machine learning classification models for the imbalanced datasets. Third, we only included nursing notes for model development as they contain the most clinical detail of daily patient care and are universally documented in all patient records. Other clinical notes, such as diagnostic reports, surgery-related reports, and discharge summaries, were not included in this study. Incorporating those notes may potentially enhance the sensitivity of the developed models, but it is likely that both the positive predictive value and overall performance will be greatly diminished. Lastly, the performance of models using clinical notes from the EMR database is contingent on the quality of reporting by nurses. Potential human errors, diverse documentation practices, and the adequacy of healthcare professionals' EMR training can influence the accuracy and reliability of the results.

## Conclusions

Detecting SSIs from large population-based cohorts is imperative for IPC surveillance programs. Our findings suggest machine learning models derived from administrative data and nursing notes in EMR text data achieved high performance and can be used to automate the process of complex SSIs detection.

### Supplementary Information

**Additional file 1**. Details of Machine Learning Model Development for SSI Detection.

## Data Availability

Due to data sharing policies of the data custodians, the dataset and developed models are not able to be made publicly available. They may be able to be shared only to researchers in Alberta with approval from the data custodians.

## Abbreviations

95% CI: 95% Confidence interval
CCI: Canadian Classification of Health Intervention
CDC: Centers for Disease Control and Prevention
DAD: Discharge abstract database
ECDC: European Centre for Disease Prevention and Control
EMR: Electronic medical records
ICD: International Classification of Diseases
IPC: Infection prevention and control
IQR: Interquartile ranges
IRR: Incidence rate ratios
MPRs: Multidisciplinary progress reports
NPV: Negative predictive value
PHN: Personal healthcare number
PPV: Positive predictive value
PR AUC: Area under the precision–recall curve
ROC AUC: Receiver operating characteristic curve
SCM: Sunrise Clinical Manager
SSIs: Surgical site infections
TF–IDF: Term frequency–inverse document frequency
THA: Total hip arthroplasty
TKA: Total knee arthroplasty
ULI: Unique lifetime identifiers
