# Supplementary material for: Development of machine learning models for the detection of surgical site infections following total hip and knee arthroplasty: a multicenter cohort study
Source: Antimicrob Resist Infect Control. 2023 Sep 2;12:88. doi: 10.1186/s13756-023-01294-0 (PMC10474760; doi:10.1186/s13756-023-01294-0)
Supplement: Supplementary file 1 — Additional file 1. Details of Machine Learning Model Development for SSI Detection. [file 13756_2023_1294_MOESM1_ESM.docx]

# Supplementary document

## Data tables used in this study.

1. **Reference table**

The reference table is used as the gold standard in this study. This tables comprises of 241 rows and 15 columns, representing the medical history of 241 different patients who underwent Total Hip/Knee Replacement surgeries at 4 different hospitals in Calgary. The information includes demographic data, procedure details, results from follow-up cultures, and readmission details. It can provide valuable insights into the rate and type of Surgical Site Infections (SSI) post-procedure, the efficacy of treatment methods, the rate of readmissions, and any potential patterns or correlations in this data. There are in total three types of SSIs were coded, which are organ-space, deep incisional and superficial incisional infections. The main data variables we used for the studies and corresponding examples are shown in Table i.

Table i. Reference table schema overview.

| **Variable** | **Example Value** |
| --- | --- |
| Provincial identifier number | e.g., 123456789 |
| Birth Date | 1971-03-21 (YYYY-MM-DD) |
| Sex | Male |
| Procedure Type | Total Knee Replacement |
| Procedure Side | Left - Unilateral |
| Procedure Date | 2015-01-09 (YYYY-MM-DD) |
| Procedure Facility | Hospital B |
| SSI Type | Superficial Incisional |
| Pt Readmitted | Yes |
| Readmission Date | 2013-01-18 |
| Current Facility | Hospital B |

1. **Discharge administrative data (DAD) table**

This dataset includes unique identifiers at both the patient level, administrative details such as chart number, and a sequence number indicating the order of events or procedures. Critical timeline markers are captured in the dates of admission and discharge, as well as the total time spent in the ICU and the entire healthcare facility. The patient's journey post-discharge is documented through a disposition code and a readmission indicator. Key demographic details are captured in the patient's date of birth (BIRTHDATE) and gender (SEX). The primary medical data, encompassing the patient's diagnoses, are recorded in the form of International Classification of Diseases 10^th^ version (ICD-10) codes, spanning up to 25 diagnoses for each patient. The specifics of these data fields may vary depending on the context and the record-keeping system used by the organization that created the dataset.

Table ii. DAD table schema overview.

| **Variable** | **Example Value** |
| --- | --- |
| Provincial identifier number | 123456789 |
| Chart number | 1234567890 |
| Sequence number | 1231231231231234 |
| Dates of admission | 2023-07-26 (YYYY-MM-DD) |
| Dates of discharge | 2023-07-26 (YYYY-MM-DD) |
| Total time spent in the ICU (days) | 0 |
| Total time spent in the facility (days) | 1 |
| Disposition | Home |
| Readmit | No |
| Birth date | 1971-03-21(YYYY-MM-DD) |
| Sex | Male |
| ICDCODE1 - ICDCODE25 (25 columns from the DAD database) | M171, J189, E119, ... |

1. **Note data**

The note data includes a unique document identifier, along with a unique identifier for each patient visit. The timestamps of document creation and the most recent update or access are meticulously recorded. Detailed timestamps of patient admission and discharge are also provided. The type of medical note, such as assessment documents, multidisciplinary progress reports, transfer notes, pain assessments, etc., are captured in both free text and standardized forms. The dataset contains abundant textual data; the original medical notes are stored in Rich Text Format (RTF), while a cleaned version of these notes, which is ideal for subsequent analysis or natural language processing tasks, is also maintained.

For each patient visit, there could be multiple types of notes recorded more than once; hence, in total, we retrieved 4,152,875 unique documents from the database.

Table iii. Note data schema overview.

| **Variable** | **Example Value** |
| --- | --- |
| Unique Document Identifier | 1231231231231234 |
| Timestamp of Document Creation | 2023-07-01 10:00:00 |
| Timestamp of Most Recent Document Update | 2023-07-01 10:15:00 |
| Unique Visit Identifier | 1231231231231234 |
| Unique Visit Code | 1231412312345 |
| Timestamp of Patient Admission | 2023-07-01 09:45:00 |
| Timestamp of Patient Discharge | 2023-07-05 10:00:00 |
| Type of Medical Note in Free Text | "Pain Assessment" |
| Type of Medical Note in Standard Form | "PAIN_ASSESSMENT" |
| Original Medical Note in Rich Text Format | "{\rtf1\ansi...}" |
| Cleaned Version of Medical Note | "Patient reports moderate pain..." |

## Supervised dataset construction

### Data linkage

Our data integration process brought together our reference table, DAD table, and note dataset through the ULI and other unique identifiers shared across these tables. This established connection provides insights into whether a patient has an SSI, leveraging coded data from the reference table. After combining these tables, patients with SSI identified by the Infection Prevention and Control (IPC) group were labeled with the respective type of SSI, while those not identified by the IPC group were labeled as "No Infection".

The resulting merged table consists of two main components: ICD-10 codes derived from administrative data and plain text sourced from note data, providing a comprehensive and versatile dataset for in-depth analysis. In the group we selected, we continued to filter the data to connect it and build a high-quality supervised dataset for administrative and note data. We only included individuals who were readmitted and who had data available from the multi-disciplinary progress report (MPR).

### Administrative data-based supervised dataset construction

Our primary objective was to construct a structured dataset with administrative data suitable for supervised learning tasks. This required a uniform representation of each patient, where the features correspond to distinct ICD-10 diagnostic codes.

To achieve this uniformity, we first created distinct columns for each possible diagnostic code. This resulted in a wide-form structure, where every diagnostic code had a dedicated column, and each patient was represented by a row. This structure ensures the same number of features for each patient, an essential requirement for many machine learning algorithms. The diagnostic codes were then binary encoded: If a patient had a particular diagnostic code, a '1' was assigned in the corresponding column. If the diagnostic code was not associated with the patient, the value was set as '0'. This transformation created a sparse matrix representation of the patients' health histories, where '1's and '0's represent the presence or absence of specific health conditions, respectively. In our experiment we extracted 1,767 unique ICD-10 codes.

In addition to the binary-encoded diagnostic codes, other essential patient information such as the unique patient identifier (ULI), the number of hours they spent in intensive care (icu_hrs), the total number of days they spent in the hospital (los), their age, and their sex, were also included in the structured dataset.

This preprocessing stage has effectively transformed our raw patient data into a structured format that can be readily ingested by machine learning algorithms for further analysis. Each row of the resulting data frame represents a comprehensive, high-dimensional profile of a patient, encapsulating both their demographic information and their health history as captured by the ICD-10 diagnostic codes. The Table iv illustrate the dataset and each row represents a different patient, identified by their ULI. This id can also help use to link to natural language dataset.

Table iv. Administrative data-based supervised dataset illustration.

| **Unique identifier** | **icu_hrs** | **los** | **age** | **sex** | **A491** | **N10** | **H353** | **U983** | **...** | **J019** | **SSI** |
| --- | --- | --- | --- | --- | --- | --- | --- | --- | --- | --- | --- |
| 123456789 | 0.0 | 4 | 66 | 0 | 0 | 0 | 0 | 0 | ... | 0 | 0 |
| 234567890 | 2.5 | 6 | 50 | 1 | 1 | 0 | 0 | 0 | ... | 0 | 1 |
| … | … | … | … | … | … | … | … | … | … | … | … |
| 456789012 | 1.5 | 3 | 80 | 0 | 0 | 0 | 1 | 0 | ... | 0 | 0 |

***Unique identifier:*** Provincial unique identifier for patient.

***icu****_****hrs****:* ICU hours represents how many hours each patient spent in the ICU.

***los***: Length of stay shows how many days each patient spent in the hospital.

**Sex**: 0 means female and 1 means male.

The columns from ***A491*** to ***J019*** represent different diagnostic codes. The ICD-10 features follow a one-hot encoding scheme, where each diagnostic code column represents a unique diagnosis, and the entries in these columns are binary. A value of '1' signifies the presence of the corresponding diagnosis in a patient, while a '0' indicates its absence.

***SSI:*** stores labels from reference table, which contain in total of four different types of labels

1: *'Deep Incisional',*

2: *'Organ-Space'*,

3: *'Superficial Incisional'*,

0: *'no infection'.*

### Textual dataset construction

Each visit of one individual patient might have more than one note. We merged all textual note into one gigantic document to represent entire information of the patient’s visit. With the merged text data, we proceed to construct textual dataset for supervised machine learning.

First, special characters removal or formatting is conducted with our custom Regular Expression (RE) based algorithm in Python programming language. The process was concluded by trimming any superfluous spaces or characters in the text to create a clean, continuous string of text.

Second, we applied a set of text normalization techniques to reduce to total number of text features. The techniques including, case folding, remove repeating terms with a custom RE-based algorithm.

Third, we applied Scispacy^[[1]](#footnote-1)^--a Python library specifically tailored for scientific and medical text processing, for medical term extraction. SciSpacy is an extension of the popular Spacy library. By applying SciSpacy to our data, we were able to extract and identify these medical terms accurately, turning raw text into structured data that is more suitable for further analysis or machine learning tasks.

Fourth, we utilized negspacy^[[2]](#footnote-2)^, a tool designed for negation detection in medical text. In healthcare records and scientific literature, expressions of negation (for example, "the patient does not have infection") are quite common. This step turned out to be crucial in avoiding any false-positive findings in the subsequent analysis and machine learning tasks.

Then, we used these extracted medical terms to build a Bag-of-Concept (BOC) feature matrix and choose TF-IDF scores for the weight. The TF-IDF score is calculated with Scikit-learn ^[[3]](#footnote-3)^library. In the feature each column represents a unique concept extracted with Scispacy, and each row representing an individual patient.

In our study, we initially extracted a large set of 125,712 BOC features from our text data. To avoid overfitting and improve computational efficiency, we conducted a subsequent feature selection process. For this, we employed a grid search strategy, which systematically evaluated various subsets of the features based on their performance in the prediction model (see Table viii). This exhaustive process ultimately helped us identify an optimal subset of 500 features (concepts). These selected features not only significantly reduced the dimensionality of our model, but also maintained or even improved the model's predictive accuracy.

Table v. Illustration of BOC.

| **Example** | **Original text (synthetic)** | **Bag-of-Concept** | **ssi** |
| --- | --- | --- | --- |
| 0 | Patient discharged after surgery; no sign of infection detected. | ['patient', 'discharged', ‘surgery'] | 0 |
| 1 | Patient came in with redness and swelling at surgical site post-hip replacement. | ['patient', 'redness', 'swelling', 'surgical_site', 'hip_replacement'] | 1 |
| 2 | Post-op patient, wound healing well with no complications, home care recommended. | ['post_op_patient', 'wound_healing', 'home_care'] | 0 |

Table vi. Illustration of textual data feature matrix.

| **Unique identifier** | **patient** | **discharged** | **surgery** | **redness** | **swelling** | **...** | **ssi** |
| --- | --- | --- | --- | --- | --- | --- | --- |
| 123456789 | 1 | 0.30 | 0.50 | 0.50 | 0 | … | 0 |
| 345678901 | 2 | 0.20 | 0 | 0.25 | 0.25 | … | 1 |
| … | … | … | … | … | … | … | … |
| 234567890 | 3 | 0 | 0 | 0 | 0 | … | 0 |

### Supervised machine learning

We built a pipeline to streamline the machine learning process. This pipeline consisted of three main steps: oversampling, and classification. The oversampling step used the Synthetic Minority Over-sampling Technique (SMOTE) to balance our dataset, which is important for training our model on an even representation of classes. The second step of the pipeline was the XGBoost Classifier, a popular machine learning algorithm known for its efficiency and performance. We implement the XGBoost ^[[4]](#footnote-4)^Python library.

To optimize our model's performance, we employed a 3-fold cross-validation grid search, utilizing the Scikit-learn library's in-built functionalities. This method examined a variety of XGBoost Classifier hyperparameter combinations— including learning rate, gamma (which manages model complexity), maximum tree depth, and the L2 regularization term (lambda). The grid search determined the ideal set of parameters based on their respective F1 scores, an established measure of model accuracy.

Moreover, we also adjusted the hyperparameters of our feature extraction tool, the vectorizer, during our optimization process. We specifically fine-tuned parameters like 'max features', setting an upper limit to the number of terms based on their frequency, and 'min_df’ and’ max df', setting the lower and upper bounds on the term frequencies across the documents in the corpus. By doing this, we ensured the extraction of the most impactful and relevant features from our text data, enhancing the quality of our input for modeling and, thus, the predictive capabilities of our model.

Table vii. Parameters and values.

| **Parameter name** | **Component** | **Values** | **Final value** |
| --- | --- | --- | --- |
| vect__max_features | TF-IDF vectorizer | All, 100 to 1000 (step 100), 1000 to 5000 (step 500), 5000 to 10000 (step 1000) | 500 |
| vect__max_df | TF-IDF vectorizer | 0.2, 0.4, 0.6, 0.8, 1 | 0.8 |
| vect__min_df | TF-IDF vectorizer | 0.2, 0.4, 0.6, 0.8, 1 | 0.4 |
| clf__gamma | XGBoost | 0.1, 0.2, 0.5, 1, 2, 4, 10 | 2 |
| clf__max_depth | XGBoost | 2, 4, 6, 8, 10, 16, 22 | 4 |
| clf__reg_lambda | XGBoost | 0.2, 0.4, 0.5, 0.8, 1 | 1 |
| clf__learning_rate | XGBoost | 0.01, 0.05, 0.1, 0.2, 0.3 | 0.2 |

To fine-tune each model according to the respective datasets, namely administrative data, text data, and a hybrid combination of both, we conducted a search for the optimal hyperparameters. This process ensured that each model was specifically tailored and fine-tuned to its corresponding dataset, maximizing the accuracy and predictive power of each model.

### Model evaluation and result Visualization

We used MedCalc^[[5]](#footnote-5)^ to calculate our evaluation metrics, including sensitivity, specificity, positive predictive value, negative predictive value. We implement AUC-ROC as well as its 95% confidence interval (CI) based on DeLong method with Python (see Table viii).

For result demonstration, we used the Matplotlib^[[6]](#footnote-6)^ library to visualize the performance of our machine learning models for text classification. Specifically, we leveraged the Precision-Recall Display function from the Scikit-Learn library, which provides a graphic representation of the trade-off between precision and recall for different thresholds.

Table viii. The Python code for calculating AUC-ROC with 95%CI.

| from sklearn.metrics import roc_curve, auc  from scipy import stats  import numpy as np  def auc_roc_95ci(y_true, y_prediction):  # y_true: the actual values from test set  # y_prediction: the predicted values from model’s prediction  # Compute ROC curve and AUC score  fpr, tpr, _ = roc_curve(y_true, y_prediction)  roc_auc = auc(fpr, tpr)  # DeLong's method for AUC-ROC 95% CI  n1 = np.sum(y_true)  n2 = len(y_true) - n1  q1 = roc_auc / (2 - roc_auc)  q2 = 2*roc_auc**2 / (1 + roc_auc)  se_auc = ((roc_auc*(1 - roc_auc) + (n1 - 1)*(q1 - roc_auc**2) + (n2 - 1)*(q2 - roc_auc**2)) / (n1*n2))**0.5  ci = stats.norm.ppf(0.975) * se_auc  lower_bound = roc_auc - ci  upper_bound = roc_auc + ci  return roc_auc, lower_bound, upper_bound |
| --- |

1. [scispacy | SpaCy models for biomedical text processing (allenai.github.io)](https://allenai.github.io/scispacy/) [↑](#footnote-ref-1)
2. [GitHub - jenojp/negspacy: spaCy pipeline object for negating concepts in text](https://github.com/jenojp/negspacy) [↑](#footnote-ref-2)
3. [GitHub - scikit-learn/scikit-learn: scikit-learn: machine learning in Python](https://github.com/scikit-learn/scikit-learn) [↑](#footnote-ref-3)
4. [XGBoost Documentation — xgboost 1.7.6 documentation](https://xgboost.readthedocs.io/en/stable/) [↑](#footnote-ref-4)
5. https://www.medcalc.org/calc/diagnostic_test.php [↑](#footnote-ref-5)
6. [Matplotlib — Visualization with Python](https://matplotlib.org/) [↑](#footnote-ref-6)
